# Regulator of G-Protein Signaling-5 Is a Marker of Hepatic Stellate Cells and Expression Mediates Response to Liver Injury

**DOI:** 10.1371/journal.pone.0108505

**Published:** 2014-10-07

**Authors:** Arya J. Bahrami, Jagadambika J. Gunaje, Brian J. Hayes, Kimberly J. Riehle, Heidi L. Kenerson, Raymond S. Yeung, April S. Stempien-Otero, Jean S. Campbell, William M. Mahoney

**Affiliations:** 1 Department of Pathology, University of Washington, Seattle, Washington, United States of America; 2 Department of Surgery, University of Washington, Seattle, Washington, United States of America; 3 Department of Cardiology, University of Washington, Seattle, Washington, United States of America; 4 Center for Cardiovascular Biology and the Institute for Stem Cell and Regenerative Medicine, University of Washington, Seattle, Washington, United States of America; Universite de Rennes 1, France

## Abstract

Liver fibrosis is mediated by hepatic stellate cells (HSCs), which respond to a variety of cytokine and growth factors to moderate the response to injury and create extracellular matrix at the site of injury. G-protein coupled receptor (GPCR)-mediated signaling, via endothelin-1 (ET-1) and angiotensin II (AngII), increases HSC contraction, migration and fibrogenesis. Regulator of G-protein signaling-5 (RGS5), an inhibitor of vasoactive GPCR agonists, functions to control GPCR-mediated contraction and hypertrophy in pericytes and smooth muscle cells (SMCs). Therefore we hypothesized that RGS5 controls GPCR signaling in activated HSCs in the context of liver injury. In this study, we localize RGS5 to the HSCs and demonstrate that *Rgs5* expression is regulated during carbon tetrachloride (CCl_4_)-induced acute and chronic liver injury in *Rgs5^LacZ/LacZ^* reporter mice. Furthermore, CCl_4_ treated RGS5-null mice develop increased hepatocyte damage and fibrosis in response to CCl_4_ and have increased expression of markers of HSC activation. Knockdown of *Rgs5* enhances ET-1-mediated signaling in HSCs *in vitro*. Taken together, we demonstrate that RGS5 is a critical regulator of GPCR signaling in HSCs and regulates HSC activation and fibrogenesis in liver injury.

## Introduction

Liver fibrosis and its sequelea of cirrhosis and hepatocellular carcinoma (HCC) are responsible for 29,000 deaths a year in the United States, making it the 12^th^ leading cause of death [1]. Injury to the liver results in a wave of cytokine mobilization [2], many of which are secreted by Kupffer cells, the liver's resident macrophages. These cytokines (*e.g.* tumor necrosis factor α (TNF- α) [3] and transforming growth factor-β1 (TGF- β) [4]) then activate HSCs, which deposit extracellular matrix (*e.g.* collagen) as part of the wound repair response. With chronic injury, ongoing inflammation and HSC activation results in accumulation of scar tissue and eventual decreased liver function [5].

In the uninjured liver, quiescent HSCs behave like pericytes [6], surrounding the endothelium of the sinusoids. However, upon injury-induced activation, HSCs become the primary collagen-producing cell in the fibrotic liver [7], [8]. HSC activation, in response to platelet derived growth factor BB (PDGF-BB) [9]–[11] and TGFβ [4], [12], is well characterized; however, G-protein coupled receptor (GPCR) signaling pathways also influence their behavior during fibrosis. AngII [13]–[15], ET-1 [16]–[20], and norepinephrine (NE) [21]–[24] have been implicated in promoting HSC activation and thus fibrosis. Therefore, modulating signaling downstream of GPCRs may represent a novel therapeutic target in liver fibrosis, potentially preventing HCC.

RGS5 is a small GTPase activating protein that inhibits Gα_q_ and Gα_i_-mediated signaling downstream of GPCRs [25]. RGS5 is primarily expressed in vascular smooth muscle cells (SMCs) and pericytes [26]–[29], and inhibits AngII- and ET-1-mediated signaling [30], [31] to regulate blood pressure [26], [32]–[34] and vascular remodeling [35], [36]. Moreover, RGS5 expression correlates with both cardiac [37] and skin [38] fibrosis, and expression is increased in multiple cancers (e.g., breast, ovarian, acute myeloid leukemia, and liver) [39]–[44].

We hypothesized that RGS5 controls liver injury via its ability to modulate GPCR-mediated signaling in activated HSCs. In this study, we localize expression of RGS5 to HSCs in the liver, and demonstrate that *Rgs5* expression is regulated in both acute and chronic liver injury. Furthermore, mice lacking RGS5 expression develop increased hepatocyte damage and fibrosis in response to carbon tetrachloride (CCl_4_). *Rgs5* expression is regulated in cultured HSCs in response to fibrogenic agonists, and ET-1-mediated signaling is potentiated in the absence of *Rgs5* expression. Taken together, we demonstrate that RGS5 is a critical regulator of GPCR signaling in HSCs, and controls HSC activation and fibrogenesis in response to liver injury.

## Materials and Methods

### Animals

*Rgs5* ^LacZ^ mice, which have a nuclear localized β-galactosidase reporter gene knocked into exon 2 of the *Rgs5* locus and mice have been backcrossed to a C57BL/6 background, were purchased (Deltagen [45]). To induce acute liver injury, mice were injected (***i.p***.) with 10 µl/g body weight CCl_4_ (Sigma-Aldrich) diluted 10% (v/v) in olive oil once. To induce chronic liver injury, mice were injected twice weekly for four weeks. Olive oil-injected animals served as controls for CCl_4_-injected mice. At the indicated time-points, mice were sacrificed using CO_2_ inhalation. The Institutional Animal Care and Use Committee of the University of Washington, which is certified by the Association for Assessment and Accreditation of Laboratory Animal Care International, approved all experiments.

### X-gal labeling

To preserve β-galactosidase activity, liver tissue was fixed in PLP (4% paraformaldehyde (PFA), 75 mM l-lysine, 10 mM sodium periodate) for 2 hours at 4°C, cryopreserved in 18% sucrose, frozen in optimum cutting temperature compound, and 5-µm cryosections were prepared for X-gal labeling or immunofluorescence (IF). LacZ activity was measured using a standard 5-bromo-4-chloro-3-indolyl-β-d-galactoside (X-gal) staining protocol [46] for 16 hours at 37°C. After washing, sections were post-fixed in 1% PFA for 5 minutes and immunolabeled for additional histologic analysis (see below).

### Measurement of relative hepatocyte cytoplasmic clearing

Approximately twenty 10× fields of H&E stained 5 µm paraffin embedded sections liver were imaged, each representing a 1.19x.89 mm area. Using the ImageJ software package, the relative cleared hepatocyte cytoplasmic area was measured. Images were automatically adjusted for contrast, converted to grey scale, and sharpened to enhance borders. The threshold tool isolated the tissue from slide background. Particle analysis included sizes 49 µm^2^ to 324 µm^2^ and circularity value between 0.3 and 1.0, yielding total particle area measured as a percentage of slide area. Data was processed by macro to remove potential bias.

### Quantification of collagen deposition

To label collagen deposition (indicative of fibrosis), 5 µm paraffin embedded tissue sections were stained with picrosirius red (365548 and P6744, Sigma) and 0.1% Fast Green (F7258, Sigma) at room temperature for 30 minutes. The relative quantification of fibrosis was measured using ImageJ. Ten 4× fields were imaged for each liver, representing a 2.95×2.21 mm area. Using the color threshold tool, red staining was isolated. The image is then converted to grey scale, and threshold is used to subtract remaining background. Measurement of the remaining area yielded the area of the slide with red staining. This was normalized to the area of the tissue (vessel lumen subtracted) for each slide to yield the percent of tissue with picrosirius red staining. Data was processed by macro to remove potential bias.

### Immunofluorescence (IF)

IF was performed using standard techniques, with liver sections incubated overnight with the following primary antibodies: rabbit anti-GFAP 1∶1000 (Z0334, Dako), chicken anti-β-gal 1∶1000 (ab9361, Abcam), rabbit anti-SMA1∶200 (ab32575 Abcam), rabbit anti-CRBP1 1∶200 (sc30106, Santa Cruz), rabbit anti-VWF 1∶200 (A0082, Dako), Rat anti-CD-31 1∶100 (553370, BD Pharmingen), and Rat anti-F4/80 1∶200 (122603, Biolegend). Immune complexes were detected with the following secondary antibodies: Alexa 488 conjugated goat anti-Rabbit IgG (A11034, Life Technologies), Alexa 488 conjugated goat anti-rat IgG (A21208, Life Technologies), Alexa 647 conjugated donkey anti-rat IgG (A21247, Life Technologies), Alexa 594 conjugated donkey anti-chicken IgG (703-516-155, Jackson Immunoresearch), antibodies. Sections were mounted with VectaShield (H-1000, Vector Labs) and imaged with a Zeiss Axiovert 200 microscope. Confocal images generated using an Olympus FV1000 confocal microscope.

### Cell Culture

The LX-2 HSC cell line was generously provided by Scott L. Friedman [47]. LX-2 cells were passaged in DMEM high glucose (Gibco/Life Technologies) supplemented with 2% FBS and penicillin-streptomycin and used between passages 10 and 20.

### HSC isolation

Mouse primary HSCs were prepared by perfusion with collagenase/pronase and density centrifugation using Optiprep (Sigma) as reported previously [48].

### siRNA Knockdown of *Rgs5* Expression

*Rgs5* was knocked down in LX-2 cells using a specific small interfering RNA (siRNA) from Life Technologies (5′-AGGAGAUUAAGAUCAAGUUTT-3′). LX-2 HSCs were transfected with Lipofectamine RNAiMAX Transfection reagent (Life Technologies) following the manufacturer's specifications. Briefly, 5×10^5^ cells were transfected with either *Rgs5*-specific siRNA (12.5 nM) or siRNA negative control (12.5 nM; Life technologies) and plated at a final density of 10^5^ cells/60 mm dish (for protein isolation) or 4×10^4^ cells/6-well dish (for RNA isolation) and grown in 2% FBS growth media. After 24 hr, the media were changed to serum-free media and cells were starved for 24 hr. Where indicated, cells were stimulated with endothelin-1 (ET-1; 100 nM; Sigma), TGFβ (5 ng/ml; R&D systems), TNFα (5 ng/ml; R&D systems), or PDGF-BB (10 ng/ml; R&D systems).

### RNA isolation and quantitative RT-PCR (qPCR)

RNA was isolated from LX-2 cells and mouse livers using the E.Z.N.A. Total RNA Kit I (Omega Bio Tek). For tissue lysates, 27 mm^3^ were homogenized in TRK lysis buffer using a stator-rotor homogenizer, as per kit instructions. cDNA was prepared by reverse transcription using the High-Capacity cDNA Reverse Transcription Kit; (Applied Biosystems). 20 ng cDNA was used in each qPCR reaction, using PerfeCTa SYBR Green FastMix (Quanta biosciences). Gene expression was calculated by the ΔΔCt method: Fold expression  = 2^−ΔΔCt^. Gene expression was normalized to Gapdh expression within each sample then normalized to individual control treatment conditions within each dataset.

### Immunoblot

Lysates were prepared from LX-2 cells 0, 10, and 20, min after ET-1 treatment by resuspending scraped cell pellets in lysis buffer [50 mM Tris·HCl (pH 8.0), 120 mM NaCl, 0.5% Igepal, 1 mM EDTA, with protease inhibitors (Calbiochem)]. After protein quantitation, 10 µg of each protein extract was separated on 10% bis-Tris gels. Proteins were transferred to PVDF and blocked with 5% nonfat dry milk (NFDM) in TBS-T (0.1% Tween). Membranes were incubated with the following primary antibodies diluted in 5% NFDM in TBS-T overnight at 4°C: 1∶1,000 phospho-p42/44 (Thr202/Tyr204; pERK) (Cell Signaling); 1∶5,000 total p42/44 (ERK) [49]. After 3× washes with TBS-T, membranes were incubated with the following secondary antibodies diluted in 5% NFDM in TBS-T at room temperature for 1 h: 1∶8,000 goat α-rabbit IgG HRP conjugate (Bio-Rad); 1∶8,000 goat α-mouse IgG HRP (Bio-Rad). After 4× washes with TBS-T, blots were incubated in ECL reagent (Super Signal West Pico, Pierce) and exposed to autoradiographic film.

### Statistics

Quantitative data were analyzed by unpaired t-test in excel. A p-value of less than 0.05 was considered significant. Where indicated, significance was analyzed using a non-parametric Mann-Whitney U test.

### Ethics statement

Mice were housed in a specific pathogen-free environment overseen by the Department of Comparative Medicine at the University of Washington with IACUC approval under protocol 4253-01.

## Results

### RGS5 is expressed in HSCs

**Figure 1:**
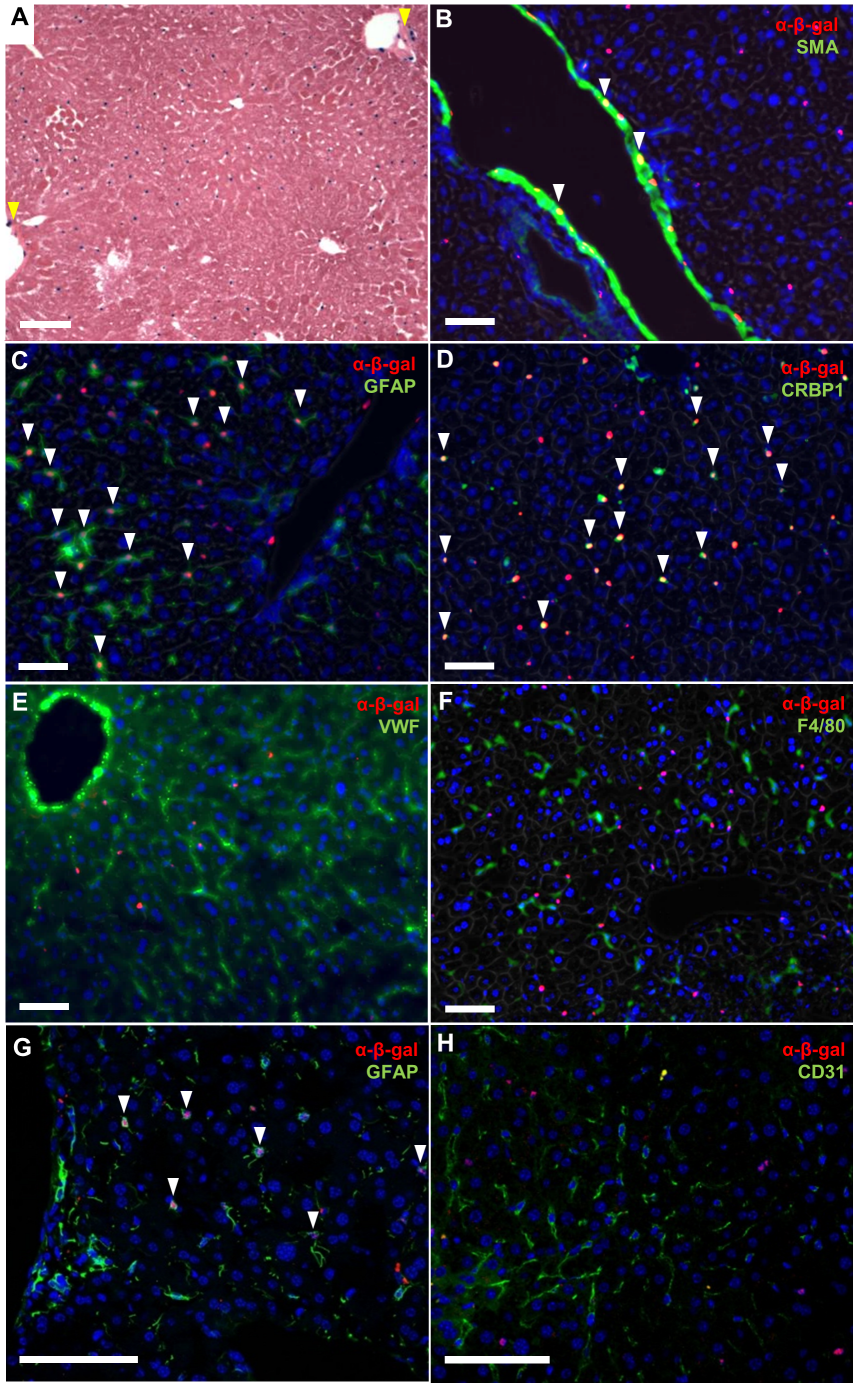
Hepatic Stellate Cells express RGS5. **A**. *Rgs5^LacZ/LacZ^* mouse liver with X-gal labeling. RGS5^+^ peri-sinusoidal cells are distributed throughout the liver. RGS5 is also expressed in a subset of SMC of the portal vein (yellow arrows). **B–G**. Immunofluorescence (IF) for cell-specific markers in the *Rgs5^LacZ/LacZ^* liver. **B**. SMA and α-β-gal show RGS5 expression in vascular SMCs, as expected (arrows). **C**. GFAP and α-β-gal IF in *Rgs5^LacZ/LacZ^* mouse liver. β-gal^+^ nuclei are visible within GFAP^+^ astrocyte-like HSC cells, localizing RGS5 expression to HSCs (arrows). **D**. CRBP1 and α-β-gal IF are co-localized in HSC of *Rgs5^LacZ/LacZ^* liver (arrows). **E**. VWF (a marker of endothelial cells) and α-β-gal are not co-localized. VWF extends through all sinusoids, while β-gal^+^ cells are sparsely distributed. **F**. F4/80 (a maker of macrophage/Kupffer cells) and β-gal^+^ cells represent distinct cell populations. **G**. Confocal image of α-GFAP IF showing co-localization of the nuclear α-β-gal (arrows). **H**. Confocal image of CD31 (a marker of endothelial cells) and α-β-gal. CD31^+^ cells have β-gal^−^ nuclei, while β-gal^+^ nuclei are not associated with CD31^+^ endothelial cells. All scale bars are 100 µm.

Using *Rgs5^LacZ^* reporter mice, we localized the expression of RGS5 in the liver by X-gal staining (Fig. 1A). β-gal^+^ cells, and therefore RGS5^+^ cells, are observed adjacent to liver sinusoids. As expected, a subset of vascular SMCs of large vessels (arrows, Fig. 1A) express both RGS5 and smooth muscle α-actin (SMA) (arrows, Fig. 1B) by IF, consistent with published findings [35]. Glial fibrillary acidic protein (GFAP) is expressed in HSCs [50], [51], and co-labeling with the GFAP and the β-gal antibody demonstrates co-localization of the RGS5 reporter and GFAP in HSCs adjacent to sinusoids (arrows, Fig. 1C). Another protein expressed in HSCs, cellular retinol-binding protein-1 (CRBP1 [52]), also co-localizes with β-gal, confirming that these cells are HSCs (arrows, Fig. 1D). Von Willebrand Factor (VWF) is expressed in endothelial cells, including those in the liver sinusoids (LSECs) [53]. IF for VWF and β-gal demonstrates that LSECs are distinct from β-gal^+^ cells (Fig. 1E); LSECs are VWF^+^, whereas the β-gal^+^ cells are sparse and evenly distributed, and do not overlap with VWF^+^ cells. Similarly, F4/80^+^ Kupffer cells and β-gal^+^ cells are also distinct, with no co-localization observed (Fig. 1F). Confocal imaging for GFAP and β-gal confirms that the nuclei of GFAP^+^ HSC are β-gal^+^ (arrows, Fig. 1G). No co-localization is observed in confocal imaging for CD31^+^, another endothelial cell marker, and β-gal^+^ cells (Fig. 1H). Finally, to verify that RGS5 expression is restricted to HSCs, we measured *Rgs5* expression in primary HSCs isolated from WT mice [48]. We found that primary HSCs express high levels of *Rgs5* relative to other non-parenchymal cell markers (Fig. S1A, B). In summary, our co-localization analyses determined that RGS5 is specifically expressed in HSCs, but not in hepatocytes, LSECs, or Kupffer cells.

### Increased RGS5 expression is associated with liver tumor and liver fibrosis

**Figure 2:**
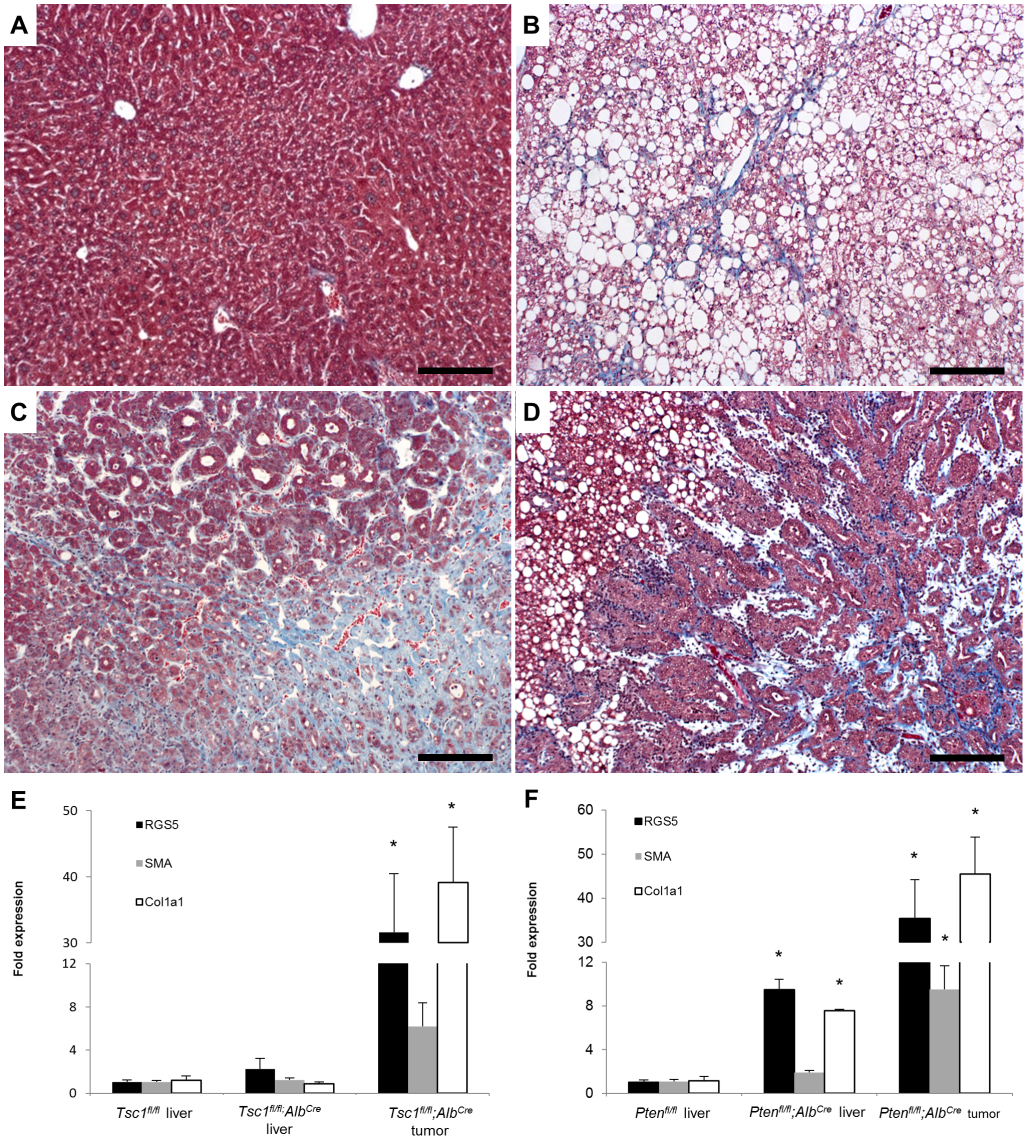
RGS5 expression is up-regulated in HCC and liver fibrosis. Sections of liver from *Tsc1^fl/fl^;Alb^Cre^* (**A**,**C**) and *Pten^fl/fl^;Alb^Cre^* (**B**,**D**) mice were stained with Masson's trichrome. **A**. *Tsc1^fl/fl^;Alb^Cre^* non-tumor liver is histologically normal. **B**. *Pten^fl/fl^;Alb^Cre^* non-tumor liver tissue is steatotic and shows collagen deposition in sinusoids (blue). **C**. *Tsc1^fl/fl^;Alb^Cre^* tumor tissue shows disorganized architecture and high levels of collagen deposition. **D**. *Pten^fl/fl^;Alb^Cre^* tumor tissue is glandular in appearance, with robust collagen deposition. Scale bars are 100 µm. **E–F**. RNA was isolated from wild-type normal tissue and matched tumor and non-tumor tissues of *Tsc1^fl/fl^;Alb^Cre^* and *Pten^fl/fl^;Alb^Cre^* **E**. *Tsc1^fl/fl^;Alb^Cre^* mice have normal RGS5, SMA, and Collagen expression in non-tumor parenchyma, and elevated expression in tumor tissue. **F**. *Pten^fl/fl^;Alb^Cre^* mice have elevated expression of RGS5 and collagen in non-tumor parenchyma and in tumors. Data is normalized to expression in wild-type liver tissue. n = 5, error bars  = SEM, * = p<0.05.

Multiple studies have demonstrated RGS5 expression in liver tumors [41], [42]. Because these tumors are associated with HSC activation, we examined RGS5 expression in two mouse models of HCC, mice with hepatocyte-specific deletion of either tuberous sclerosis complex 1 (*Tsc1*) or phosphatase and tensin homolog (*Pten*). The loss of either of these genes results in disruption of the (PI3K)/AKT/mTORC1 pathway, leading to HCC or cholangiocarcinoma [54]. First we performed trichrome staining on *Tsc1^fl/fl^;Alb^Cre^* and *Pten^fl/fl^;Alb^Cre^* liver sections spanning HCCs and adjacent non-tumor liver. *Tsc1^fl/fl^;Alb^Cre^* mice had collagen deposition in tumors (Fig. 2C), but not in adjacent non-tumor liver (Fig. 2A), while *Pten^fl/fl^;Alb^Cre^* mice had significant trichrome staining in both tumor and non-tumor liver (Fig. 2B, D). When we macro-dissected tumors from these mice and performed qPCR for *Rgs5* expression (Fig. 2E, F), we found that *Rgs5* expression mirrored collagen deposition: 1) *Rgs5* is elevated in fibrotic *Tsc1^fl/fl^;Alb^Cre^* tumors but not adjacent non-fibrotic liver; 2) *Rgs5* is elevated in both tumor and surrounding liver (both fibrotic) in *Pten^fl/fl^;Alb^Cre^* mice. The up-regulation of *Rgs5* expression in fibrotic liver tissue suggests that *Rgs5* expression may be associated with HSC activation, which occurs both in liver injury and in HCC [55].

### RGS5 expression is up-regulated in acute CCl_4_ injury

**Figure 3:**
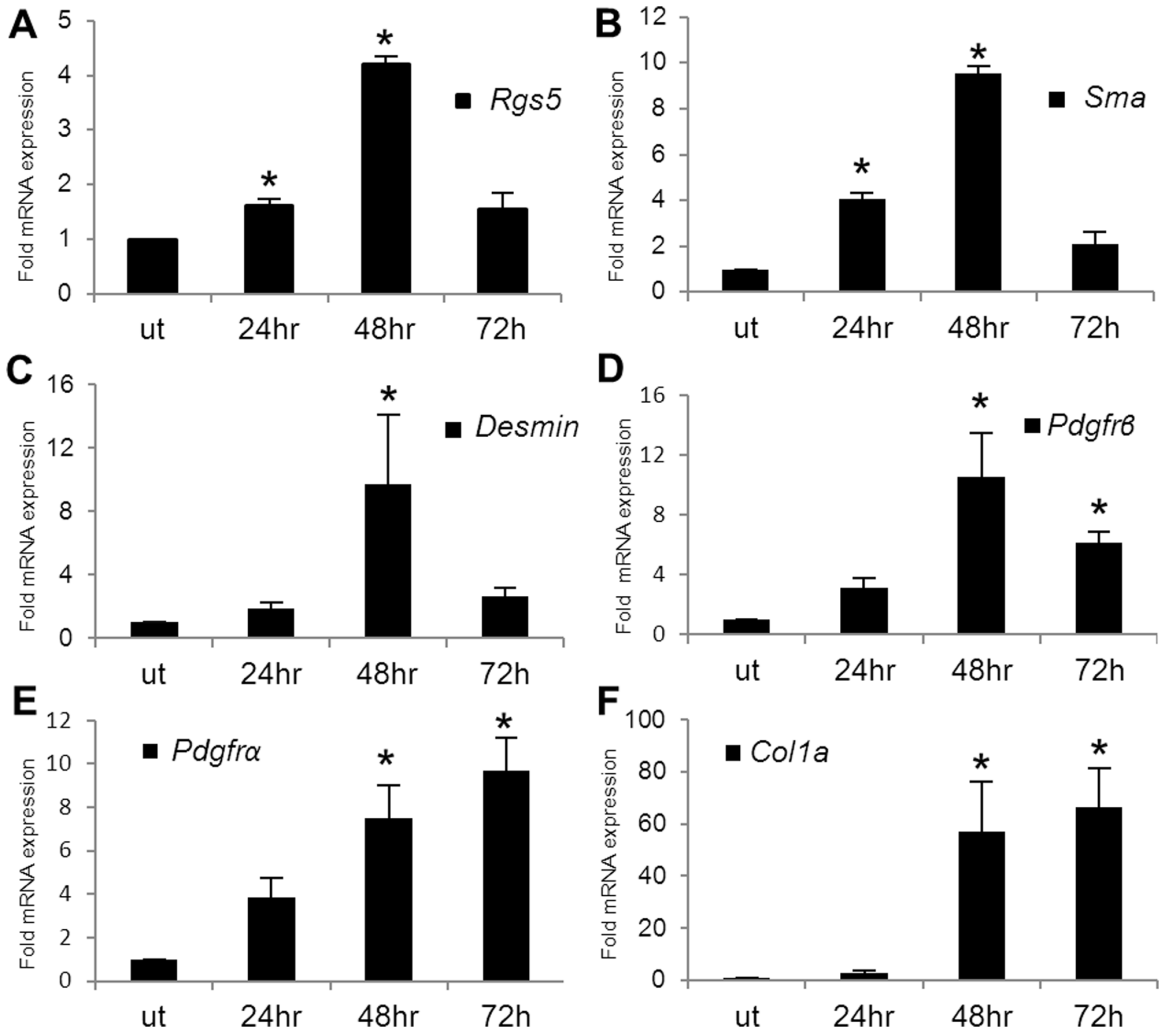
RGS5 expression is up-regulated in acute liver injury. C57BL/6 mice were injected i.p. with 10 µl/g body weight 10% CCl_4_ diluted in olive oil 10% (v/v). Livers were collected at 24, 48, 72 hr post injection. RNA was isolated and expression of HSC activation markers was determined by qPCR. **A**. RGS5 expression is elevated from 24 to 72 hr post injury, peaking at 48 hr. Expression of **B**. SMA, **C**. Desmin, and **D**. PDGFRβ is up-regulated at 24 and 48 hours post injury. **E**. PDGFRα and **F**. Col 1a are up-regulated 48 hours post injury and remain high at 72 hr. Data is normalized to expression in untreated (ut) samples. n = 3–5, error bars  = SEM, * = p<0.05.

Acute injection of CCl_4_ leads to hepatocyte death and a subsequent injury response [56]–[58]. We found that acute CCl_4_-induced injury induces a 4-fold up-regulation of *Rgs5* mRNA expression 48 hours after injection (Fig. 3A). Elevated *Rgs5* expression correlates with the increased expression of additional markers of HSC activation and fibrosis in the murine liver, including *Sma* (Fig. 3B), *Desmin* (Fig. 3C), and *Pdgfrβ* (Fig. 3D) [15], [59], which also peak at 48 hours post injury. *Pdgfrα* (Fig 3E) and *Col1a* (Fig 3F) are up-regulated at 48 hours, but peak at 72 hours post injury. Taken together, these data support the hypothesis that RGS5 is associated with fibrosis and HSC activation in both tumor and non-tumor associated fibrosis, and that the observed up-regulation of RGS5 expression in fibrotic liver tumors [41], [42] may be due to tumor associated activated HSCs.

### RGS5 expression is regulated in response to inflammatory and profibrotic stimuli *in vitro*

**Figure 4:**
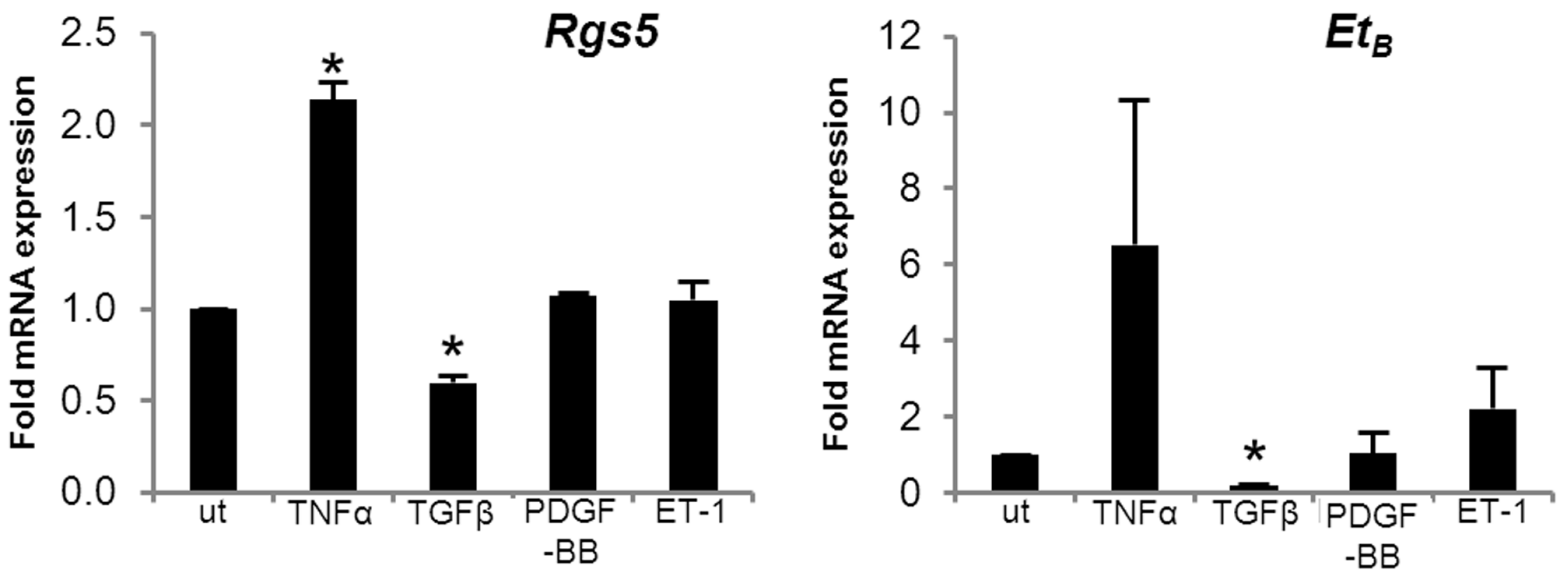
RGS5 expression is regulated by profibrotic cytokines in concert with ET_B_. LX2 HSCs were treated with TNFα (5 ng/ml), TGFβ (5 ng/ml), PDGF-BB (10 µM), and ET-1 (100 nm) for 24 hr. RNA was collected for qPCR analysis of RGS5 and ET_B_ expression. Both RGS5 and ET_B_ are up-regulated by TNFα stimulation and down-regulated by TGFβ stimulation. RGS5 and ET_B_ expression are correlated, responding similarly to the same stimuli. n = 3, error bars  = SEM, * = p<0.05.

HSCs are activated in response to the growth factor and cytokine milieu released during the response to hepatocyte injury, including TNFα [60], TGFβ [61], PDGF-BB [62], and ET-1 [63]. To determine whether RGS5 expression is affected by any of these factors, we stimulated LX-2 cells and assayed the expression of *Rgs5* by qPCR. *Rgs5* and endothelin receptor B (ET_B_) expression were up-regulated by TNFα stimulation but inhibited by TGFβ stimulation (Fig. 4) ET_B_ is a marker of HSC activation [64], and activated receptors are rapidly internalized, serving as a sink for ET-1 agonists [65]. The simultaneous co-regulation of RGS5 and ET_B_ expression makes sense, as ET_B_ sequestering ET-1 agonist and RGS5 inhibiting Gα_q_-mediated signaling both result in the blockade of ET-1-mediated signal transduction. Regulation of RGS5 in response to cytokines released after hepatocyte injury could enable tunable control of ET-1-mediated signaling in HSCs.

### RGS5^+^ HSCs participate in the response to hepatic injury

**Figure 5:**
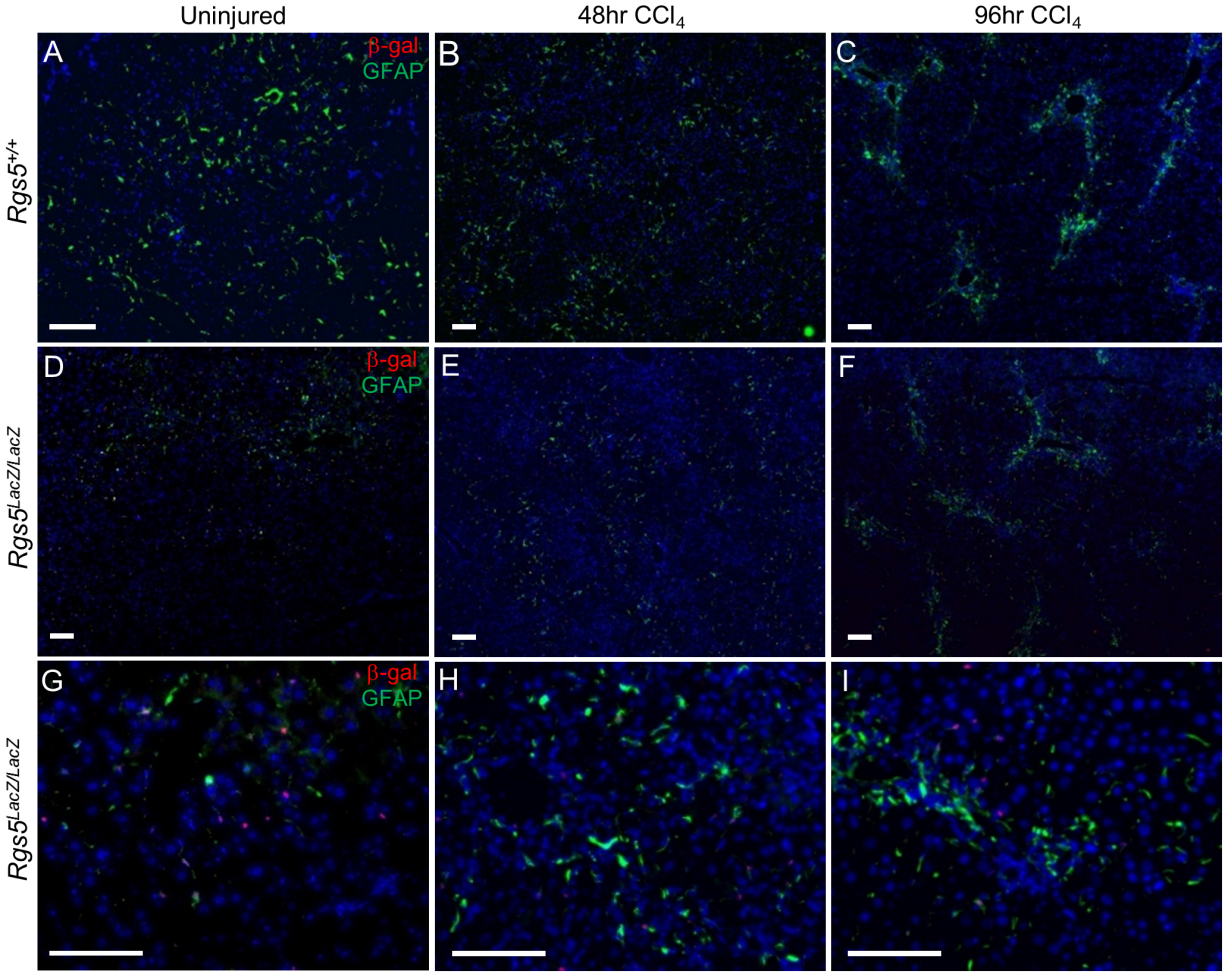
RGS5^+^ HSCs participate in the response to acute hepatic injury. Anti-GFAP and anti-β-gal immunofluorescence were used to localize HSCs in acutely injured liver tissue. **A–F**. Low magnification images. **G–I**. High magnification of *Rgs5^LacZ/LacZ^* **A,D** Uninjured liver tissue from *Rgs5^+/+^* and *Rgs5^LacZ/LacZ^* mice show sparse HSCs distributed throughout the liver. High magnification in uninjured *Rgs5^LacZ/LacZ^* shows HSCs are GFAP^+^ and β-gal^+^. **B,E**. At 48 hours post injury, HSCs are concentrated in the necrotic foci surrounding the central veins. β-gal^+^ cells (**E,H**) are associated with GFAP^+^ cells. **C,F** At 96 hours post injury, HSCs are tightly clustered at the foci of injury. **I**. β-gal^+^ cells are GFAP^+^. All scale bars are 100 µm.

As stated above, RGS5 expression is up-regulated in both genetically-induced HCC (Fig. 2) and acutely in response to CCl_4_-induced liver injury (Fig. 3). To investigate the role of RGS5 in mediating HSC activation and liver fibrosis, we induced liver injury (CCl_4_ model, as above) in *Rgs5^LacZ/LacZ^* mice to assess the changes in RGS5 cellular expression over time during injury. To identify RGS5^+^ HSCs during the injury response, we co-localized expression of GFAP and β-gal by IF. In uninjured liver tissue, HSCs are sparsely distributed throughout the liver of both *Rgs5^+/+^* and *Rgs5^LacZ/LacZ^* mice (Fig. 5A,D). However, 48 hr post CCl_4_ injury, HSCs are present in the necrotic foci (Fig. 5B). β-gal^+^ HSCs are clustered in the necrotic foci, and are scarce in the uninjured parenchyma (Fig. 5E). At 96 hours, HSCs are tightly clustered at the foci of injury and are rare in the parenchyma (Fig. 5C, F). Co-localization analysis (Fig. S2) shows 75% of β-gal^+^ cells are GFAP^+^, and that this ratio does not significantly change over the course of injury. The expression of RGS5 remains localized to GFAP^+^ HSCs before and during injury (Fig. 5G–I), and its up-regulation correlates with expression of HSC activation markers (desmin and SMA) [15], [59], peaking at 48 hr post injury (Fig. 3). Since RGS5 functions to inhibit GPCR signaling [25], the up-regulation of RGS5 mRNA in HSCs during the liver injury response suggests a role in controlling GPCR mediated HSC activation.

### RGS5 deficient mice have increased hepatocyte damage in response to acute liver injury

**Figure 6:**
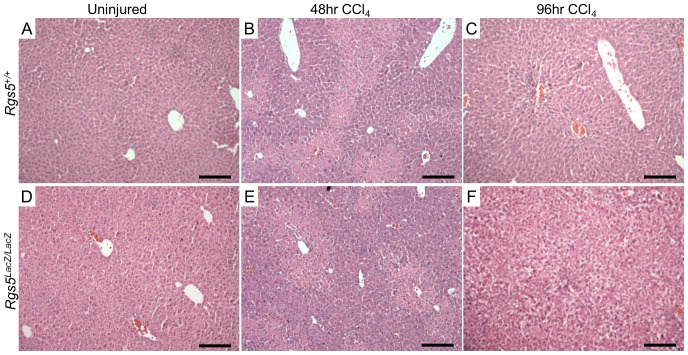
*Rgs5^LacZ/LacZ^* mice have disrupted hepatocyte morphology after injury. Acute CCl_4_-induced injury in *Rgs5^+/+^* mice (**A–C**) and *Rgs5^LacZ/LacZ^* (**D–F**). **A,D**. Uninjured mice are histologically normal. **B**,**E** At 48 hr post CCl_4_ injection, foci of necrosis are visible central veins in both *Rgs5^+/+^* and *Rgs5^LacZ/LacZ^* mice. **C,F**. At 96 hr post injury, clearance of necrotic hepatocytes is underway and infiltrating cells remain at the site of injury. **F**. In *Rgs5^LacZ/LacZ^* mice, hepatocytes throughout the liver have cleared cytoplasm. Scale bars are 100 µm.

**Figure 7:**
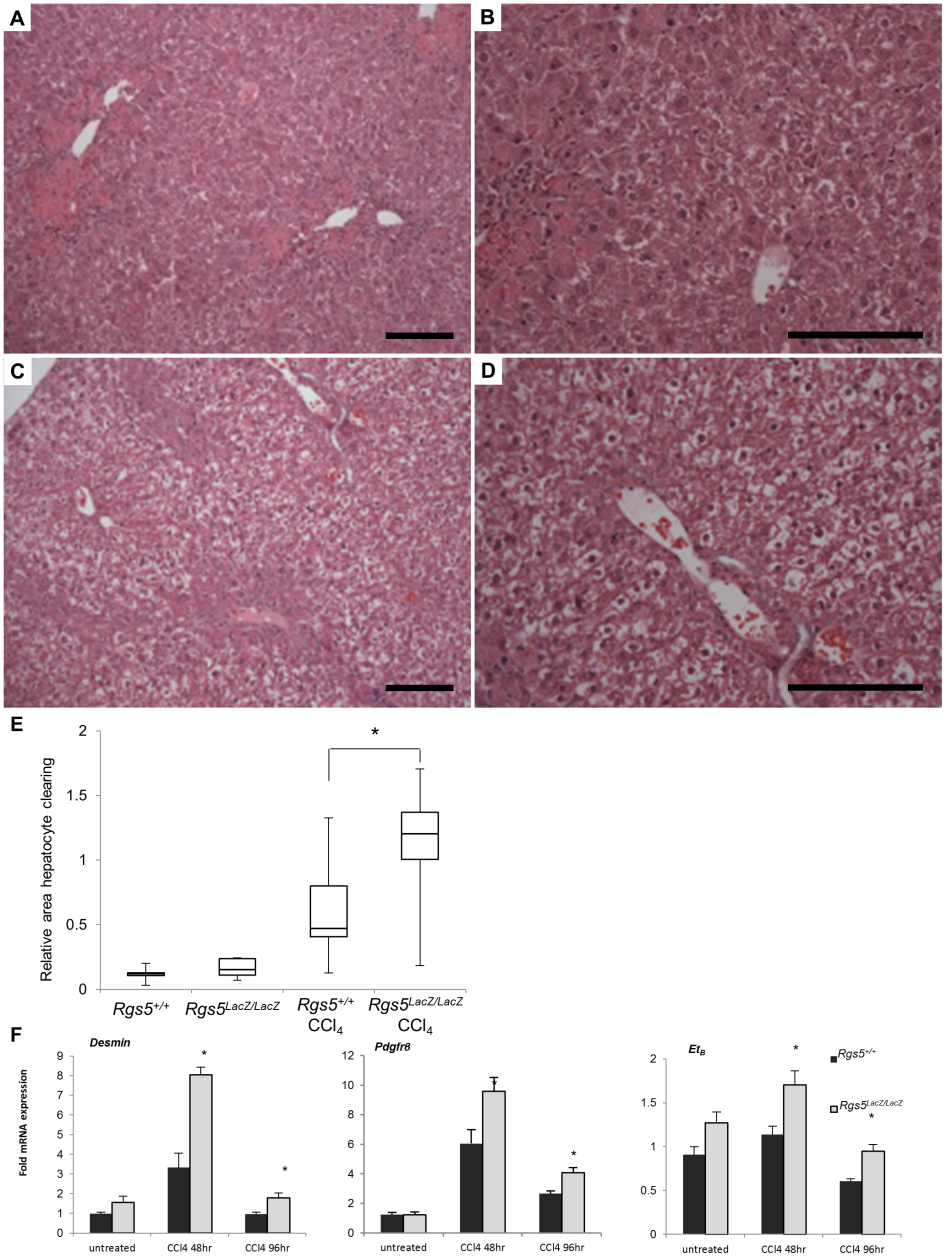
*Rgs5^LacZ/LacZ^* mice have increased liver injury and HSC activation following acute injury. Livers from *Rgs5^+/+^* and *Rgs5^LacZ/LacZ^* were collected at 96 hr following a single CCl_4_ injection. **A**. and **B**. H&E stain of *Rgs5^+/+^* mice recover normally from acute injury. Foci of necrosis are centered on the central veins. **B**. Hepatocytes appear normal. **C**. and **D**. *Rgs5^LacZ/LacZ^* livers have extensive ballooning of hepatocytes throughout the liver. Foci of necrosis are present. **D**. *Rgs5^Lacz/LacZ^* hepatocytes show cleared cytoplasm and centralized nuclei in hepatocytes distant from necrotic foci. Scale bars are 100 µm. **E**. Quantification of damaged hepatocyte area reveals a significant increase in ballooning in *Rgs5^Lacz/LacZ^* mice compared to *Rgs5^+/+^* mice (n = 8–10; error bars  = SEM; * = p<0.05) **F**. mRNA expression of markers of HSC activation (Desmin, PDGFRβ, and ET_B_) are elevated in *Rgs5^Lacz/LacZ^* mice compared to *Rgs5^+/+^* mice following acute CCl_4_ injury. n = 4–6, * = p<0.05 by Mann-Whitney U test.

Given the induction of RGS5 expression after CCl_4_ injection, we next used *Rgs5^LacZ/LacZ^* mice to assess the functional consequences of loss of RGS5 expression. Uninjured liver appears histologically normal in *Rgs5^LacZ/LacZ^* mice (Fig. 6A,D). At 48 hr post injury, foci of necrosis are visible around the central veins (Fig. 6B,E). At 96 hr post injury, necrotic hepatocytes undergo clearance and infiltrating cells remain at the site of injury (denoted by arrow in Fig. 6C&F). In *Rgs5^LacZ/LacZ^* mice, necrotic foci are similar to wild-type mice. At 96 hr post injury, the foci of necrosis are diminished; however, hepatocytes throughout the liver have cleared cytoplasm and have a ballooned appearance (Fig. 6F). To determine if *Rgs5^LacZ/LacZ^* mice are more susceptible to CCl_4_-induced liver damage than wild-type mice, we performed careful morphological analysis of H&E stained sections of liver tissue 96 hr after injury. *Rgs5^+/+^* mice have comparatively normal hepatocytes (Fig. 7A, B). In contrast, *Rgs5^LacZ/LacZ^* mice demonstrated widespread hepatocyte ballooning (Fig. 7C, D). Furthermore, in *Rgs5^LacZ/LacZ^* mice, the hepatocytes are characterized by cleared cytoplasm and central nuclei (Fig. 7D). Quantification of cleared hepatocyte area, using ImageJ, confirms a significant increase in injured hepatocyte area in *Rgs5^LacZ/LacZ^* mice (Fig. 7E). Further, by analyzing the liver/body weight ratio in *Rgs5^+/+^* compared to *Rgs5^LacZ/LacZ^* mice, we observe injured *Rgs5^LacZ/LacZ^* livers are larger than uninjured livers, while *Rgs5^+/+^* liver/body weight ratios do not show an injury-induced increase in size (Fig. S3). Oil red-O staining shows that the cleared hepatocytes do not contain lipids (Fig. S4), nor accumulated glycogen (Fig. S5). TUNEL staining shows no difference in apoptosis between the genotypes, and no association with cleared hepatocytes (Fig. S6).

We propose that in the absence of RGS5 expression in HSCs, HSCs are increasingly sensitive to GPCR agonists, such as ET-1 and AngII, which contribute to HSC activation, portal hypertension, and the severity of fibrosis [14]–[19]. Excessive ET-1 or AngII signaling in *Rgs5^LacZ/LacZ^* mice may induce inappropriate or excessive activation of HSCs, causing increased stress on hepatocytes during injury. Therefore, we compared the mRNA expression of HSC activation markers in *Rgs5^+/+^* and *Rgs5^LacZ/LacZ^* mice in acutely injured liver tissue. Expression of desmin, endothelin-1 receptor B (ET_B_), and PDGFRβ [15], [59] is increased in *Rgs5^LacZ/LacZ^* mouse liver tissue relative to *Rgs5^+/+^* littermates at 48 hr post CCl_4_ injury (Fig. 7F). The observed increase in expression of HSC activation markers may be attributed to an increase in HSC proliferation or an increase in HSC activation throughout the liver of *Rgs5^LacZ/LacZ^* mice. Taken together these data suggest RGS5 controls HSC activation in acute liver injury.

### RGS5 suppresses ET-1 signaling in HSCs

**Figure 8:**
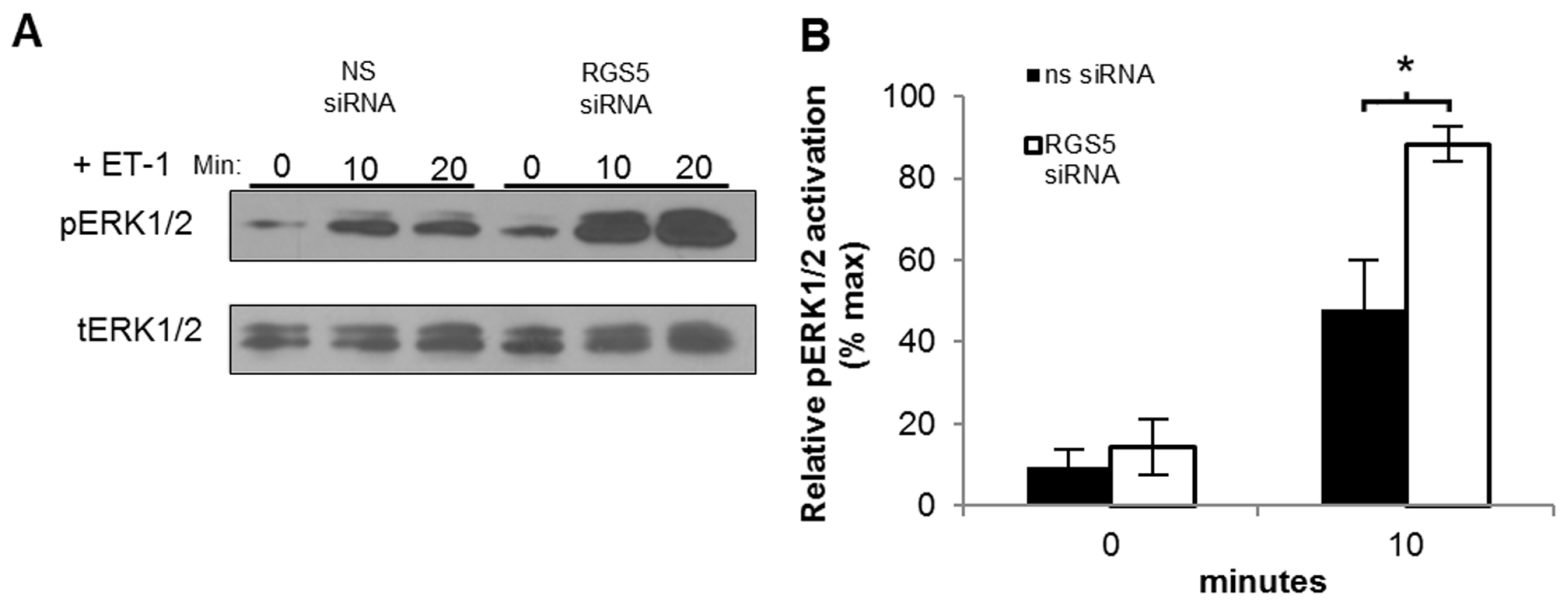
Knock-down of RGS5 expression enhances endothelin-1-mediated signaling in LX-2 HSCs. LX2 cells were treated with *Rgs5* siRNA or non-specific siRNA for 24 hours, then stimulated with 100 nM ET-1 for the indicated times. Whole cell protein extracts were isolated and analyzed by Western blot. **A**. A representative immunoblot against pERK1/2 demonstrates increased ET-1-mediated signaling in the absence of RGS5 expression; tERK serves as loading control. **B**. Quantitation of densitometry of (**A**) n = 7, error bars  = SEM, * = p<0.05.

As stated above, the increased hepatocyte injury and fibrosis observed in the *Rgs5^LacZ/LacZ^* mouse may be due to excessive signaling through GPCRs, since RGS5 inhibits Gα_q_ signal transduction [25]. To elucidate the role of RGS5 in HSCs during activation and fibrosis, we utilized a human HSC cell line (LX-2 HSCs [47]) to investigate RGS5-mediated ET-1 signaling *in vitro*. LX-2 cells are transfected with RGS5 siRNA or non-specific siRNA, stimulated with ET-1, and the activation of MAPK signaling was determined. As shown in Fig. 8A and 8B, knock-down of RGS5 expression significantly increases ERK1/2 phosphorylation in response to ET-1 in LX-2 HSCs. Therefore, in the context of increased ET-1-mediated signaling during liver injury, the absence of RGS5 expression could further exacerbate fibrogenic signaling.

### RGS5 expression is up-regulated in chronic liver injury

**Figure 9:**
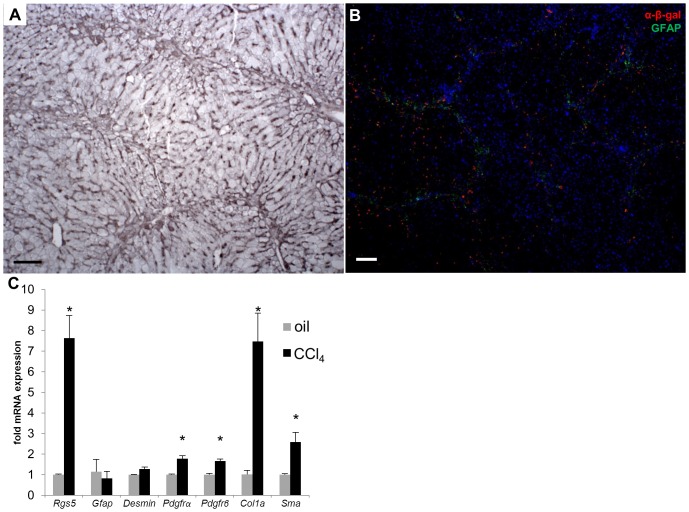
RGS5 expression is up-regulated with HSC activation in chronic liver injury. *Rgs5^LacZ/LacZ^* mice were chronically injected with CCl_4_ (or oil), twice weekly for 4 weeks. RNA was isolated from whole liver and analyzed by qPCR for expression of Rgs5 and HSC activation markers. **A**. Chronic CCl_4_ treated *Rgs5^LacZ/LacZ^* dab labeled with anti-SMA shows activated HSCs. **B**. *Rgs5^LacZ/LacZ^* liver immunolabeled with α-β-gal and α-GFAP antibodies. GFAP^+^ HSCs are visible along fibrotic septa bridging the portal veins. β-gal^+^ HSCs are visible *in Rgs5^LacZ/LacZ^* (**B**) around the fibrotic septa. Scale bars are 100 µm. **C**. qPCR of *Rgs5^+/+^* liver RNA shows Rgs5 is up-regulated in chronic CCl_4_ injury. Collagen 1α expression is elevated and multiple HSC activation markers (PDGFRα, PDGFRβ, and SMA) are increased relative to oil-injected mice. Data is normalized to oil injected mice. n = 6; error bars  = SEM; * = p<.05.

While acute injury models provide insight to the HSC response to damage, HSCs produce detectable fibrosis and scarring during chronic liver injury. Chronic exposure to CCl_4_ in mice recapitulates the fibrosis observed in human chronic liver disease [58]. *Rgs5^+/+^* and *Rgs5^LacZ/LacZ^* mice were repeatedly injected with CCl_4_ over a period of 4 weeks. In damaged *Rgs5^LacZ/LacZ^* liver tissue, SMA expression localizes activate HSCs in fibrotic septa (Fig. 9A). HSCs (GFAP^+^, βGAL^+^) were associated with fibrotic septa bridging the portal veins, near the sites of collagen deposition (Fig. 9B). Interestingly, the β-gal reporter is limited to these GFAP expressing cells in the chronically injured liver. RGS5 mRNA expression is increased 8-fold in the chronic CCl_4_ injured liver (Fig. 9C), while HSC activation markers desmin, SMA, PDGFRα, and PDGFRβ are elevated, yet below the levels observed in acute CCl_4_ injury (Fig. 3). Elevated expression of RGS5 in HSCs during the chronic CCl_4_ injury indicates an ongoing role during fibrosis, while classic markers of HSC activation have subsided, suggesting RGS5's function may extend beyond controlling HSC activation.

### RGS5 deficient mice have increased liver fibrosis during chronic injury

**Figure 10:**
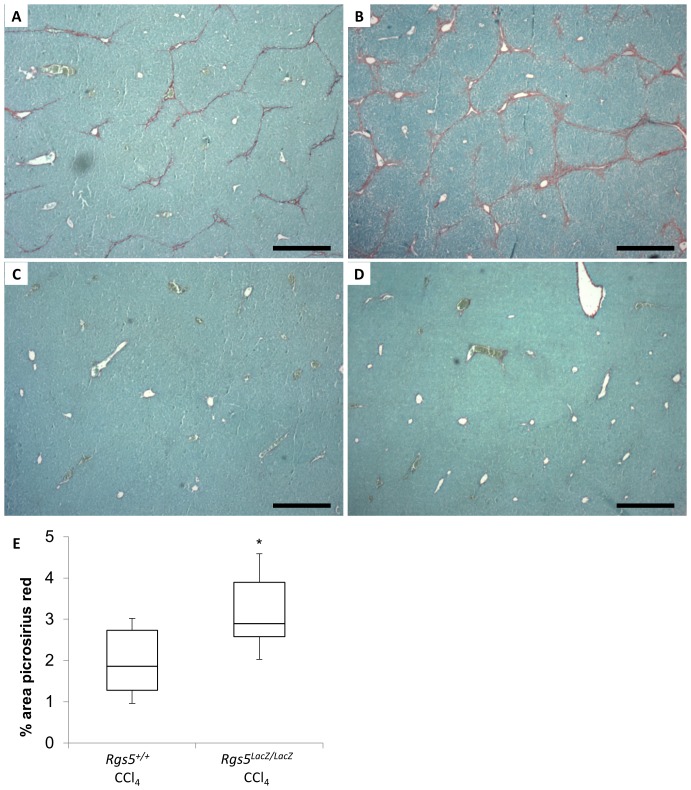
*Rgs5^LacZ/LacZ^* mice have increased fibrosis during chronic liver injury. Mice were injected with CCl_4_ (or oil), twice weekly for 4 weeks. Formalin fixed, paraffin embedded liver sections were stained with picrosirius red to assess fibrosis. **A**. *Rgs5^+/+^* mice show periportal fibrosis and bridging fibrosis between portal veins. **B**. *Rgs5^LacZ/LacZ^* mice show severe periportal fibrosis bridging between portal veins and surrounding lobules. **C**. *Rgs5^+/+^* and **D**. *Rgs5^LacZ/LacZ^* oil injected mice have minimal fibrosis. Scale bars are 500 µm. **E**. ImageJ quantitation of total picrosirius red staining in chronic CCl_4_ injury shows significantly more fibrosis in *Rgs5^LacZ/LacZ^* mice, compared to *Rgs5^+/+^* mice. n = 6, * = p<0.05 by Mann-Whitney U test.

In the acute liver injury model, *Rgs5^LacZ/LacZ^* mice were characterized by an increase in ballooning of hepatocytes (Fig. 7E) and increased liver/body weight ratio (Fig. S3), as compared to *Rgs5^+/+^* mice. To determine whether this phenotype persists in the chronic liver injury setting, hepatic fibrosis was visualized and quantified using picrosirius red. Fibular collagen is stained red, and the relative degree of fibrosis is quantitated by ImageJ (Fig. 10 A–D). Chronic CCl_4_-treated *Rgs5^+/+^* mice develop scar tissue bridging between portal veins (Fig. 10A), while oil-treated control mice are histologically normal, with minimal picrosirius red staining in *Rgs5^+/+^* (Fig. 10C) and *Rgs5^LacZ/LacZ^* (Fig. 10D). In contrast, chronic CCl_4_-treated *Rgs5^LacZ/LacZ^* mice develop severe fibrosis, as demonstrated by fibrotic septa bridging between portal veins and encircling hepatic lobules (Fig. 10B). Quantification of picrosirius red staining reveals significantly increased fibrosis in *Rgs5^LacZ/LacZ^* mice relative to *Rgs5^+/+^* mice (Fig. 10E). Taken together with the finding that RGS5 is up-regulated in chronic injury (Fig. 9A), the increase in fibrosis in *Rgs5^LacZ/LacZ^* mice suggests a RGS5-dependent role in controlling HSC-mediated collagen deposition.

## Discussion

In this study, we have identified RGS5 as a marker of HSCs and a regulator of GPCR-mediated signaling in the liver. Up-regulation of *Rgs5* expression correlates with HSC activation during liver injury, and *Rgs5* is highly expressed in the fibrotic liver. RGS5 deficient mice develop more severe liver injury following acute CCl_4_ exposure, and increased fibrosis after chronic CCl_4_ administration. *In vitro*, profibrotic and pro-inflammatory mediators regulate *Rgs5* expression in HSCs, and RGS5 knockdown in HSCs resulted in increased ERK1/2 signaling in response to ET-1, a possible mechanism for its effects in the liver. Taken together, these data suggest that the regulation of RGS5 in HSCs allows for tunable sensitivity to ET-1 signaling following hepatic injury. Loss of RGS5 disrupts this fine level of control, leading to increased HSC activation, hepatic injury, and fibrosis.

The *Rgs5^LacZ^* reporter mouse provides a robust and sensitive method to specifically localize RGS5 expression *in vivo*. Co-localization of β-galactosidase activity and traditional HSC markers (GFAP [50] and CRBP1 [66]) in these mice establishes RGS5 as a marker of HSCs in the liver. RGS proteins have been implicated in the progression of HCC [42], [67], and an earlier study localized hepatic *Rgs5* expression to endothelial cells in HCC [41] using *in situ* hybridization (ISH). However, the nature of ISH makes precise localization and discrimination between adjacent cells difficult. IF labeling of endothelial cells with an antibody to detect VWF confirms that RGS5^+^ HSCs are distinct from endothelial cells. A recent publication correlates RGS5 expression with increased vascular invasion, tumor recurrence, and decreased survival in patients with HCC [42]. However, HSCs are the major stromal cell in the tumor microenvironment, and promote HCC proliferation and invasion [68]–[70]. The correlation of increased RGS5 expression with decreased survival may reflect the level of HSC activation within the tumor, therefore predicting tumor metastasis and proliferation. High RGS5 expression has also been found in select patient-derived HCC cell lines, suggesting expression by transformed hepatocytes. These data should be interpreted with caution, however, as high passage number in culture conditions containing serum and associated growth factors may select for a phenotype that does not reflect *in vivo* expression. We therefore propose that the specific co-localization of β-gal expression (and therefore RGS5 expression) with markers of HSCs, and not with markers of other non-parenchymal cells, establishes RGS5 as a marker of HSCs.

We observed that RGS5 deficient mice had widespread hepatocyte clearing after a single CCl_4_ injection, while this phenotype was not observed in *Rgs5^+/+^* mice (Fig. 7). The hepatocyte cytoplasmic clearing observed after acute CCl_4_ treated *Rgs5^LacZ/LacZ^* mice is similar to ballooning observed in human cases of non-alcoholic fatty liver disease (NAFLD). Conflicting evidence suggests RGS5 plays a role in maintaining body weight and steatosis, with one group reporting that *Rgs5^−/−^* mice exhibit spontaneous hepatic steatosis and obesity [71] while another study demonstrates *RGS5^−/−^* mice have low body weight [34]. Although RGS proteins have been implicated in the control of hepatic fatty acid oxidation [72] and homeostasis [73], we did not observe any differences in baseline mouse body weight or oil red-o staining (Fig. S3, S4) after CCl_4_ injection, indicating that the ballooning is not due to lipid accumulation. The differences in our findings may be due to differences in mouse strains or housing conditions used in the different institutions, and the overall effects of RGS5 expression on body weight are unclear. Glycogen storage can also induce ballooning in hepatocytes, but periodic acid-schiff staining showed no difference between *Rgs5^LacZ/LacZ^* mice and wild type littermates (Fig. S5). Hepatocyte swelling reflects reversible cell injury [74] in *Rgs5^LacZ/LacZ^* mice, and it has been suggested that ballooning is protective in hepatotoxicity [59], [75]. To explore this possibility, TUNEL staining was conducted to determine whether ballooned hepatocytes were undergoing apoptosis. However, there was minimal staining in hepatocytes 96 hrs after CCl_4_ injection, and no difference was observed between *Rgs5^LacZ/LacZ^* mice and wild type littermates (Fig. S6). We similarly did not appreciate a difference in liver necrosis between the genotypes, indicating that the centrilobular zonal expression of cytochrome p450, and thus CCl_4_ metabolism, is unchanged in *Rgs5^LacZ/LacZ^mice*. Further, *Rgs5^LacZ/LacZ^* mice survive repeated doses of CCl_4_ in the chronic liver injury model, suggesting that the hepatocyte swelling is reversible.

Feathery hepatocyte degeneration is commonly observed in cholestasis [76] and is characterized by enlarged periportal hepatocytes with a flocculent cytoplasm. Intrahepatic cholestasis due to endotoxemia or drug toxicity can induce feathery degeneration; however, we did not observe pigmented hepatocytes in *Rgs5^LacZ/LacZ^* mice, and the hepatocyte swelling was equally distributed throughout the liver lobule. Whether hepatocyte swelling enhances hepatocyte survival or is simply a surrogate for more severe injury, *Rgs5^LacZ/LacZ^* mice show extensive hepatocyte swelling after a single injection of CCl_4_, while *Rgs5^+/+^* mice have normal histology.

Potential mechanisms for the increased injury observed in *Rgs5^LacZ/LacZ^* mice include portal hypertension or increased activation of HSCs. ET-1 and AngII-mediated sinusoidal constriction by HSCs induces portal hypertension [19], [77], [78]; loss of RGS5 expression enhances GPCR signaling [31] in response to these agonists, and may thus result in sinusoidal constriction. AngII infusion exacerbates fibrosis in rodent models of fibrosis [14] and ET-1 infusion increases HSC contraction and portal hypertension [79]. These effects are similar to what is observed in the Rgs5^LacZ/LacZ^ mice during CCl_4_ injury. Loss of VEGF induces sinusoidal capillarization, portal hypertension and HSC activation [80] in mice. However no parenchymal injury was observed, invalidating portal hypertension as a potential mechanism.

The increased hepatocyte injury in *Rgs5^LacZ/LacZ^* mice may be due to enhanced HSC activation via GPCR signaling. ET-1-mediated control of HSC activation has been demonstrated in previous studies, in which pharmaceutical inhibition of AngII and ET-1 reduces HSC activation *in vitro* [13], [17] and in rat models of cirrhosis [15], [19]. Our findings of increased expression of HSC markers (desmin, ET_B_, and PDGFRβ) in *Rgs5^LacZ/LacZ^* mice are consistent with this hypothesis, as removal of ET-1 inhibition would enhance expression of markers of HSC activation. ET-1 induced TGFβ secretion [81], [82] would be expected to increase in the absence of RGS5 expression, further activating HSCs and contributing to enhanced fibrosis after chronic CCl_4_ administration. This hypothesis could be further confirmed by rescue of the RGS5-null phenotype through ET-1 antagonism. For instance, the ET_A_ antagonist BQ-123 and the ET_B_ antagonist BQ-788 could be administered to *Rgs5^LacZ/LacZ^* mice during CCl_4_ injury, and would be expected to mitigate the hepatocyte swelling and increased fibrosis that we found in these mice.

RGS5-deficient mice exhibit enhanced arterial hypertrophy and perivascular fibrosis in a hypertension-induced vascular injury model [83]. This pathogenic remodeling was attributed to enhanced MEK/ERK and Rho kinase (ROCK) signaling via increased AngII-induced Gα_q_ signaling in RGS5-null mice. Enhanced ERK activity due to RGS5 knock-down has been previously reported in aortic SMCs [31], and we observed enhanced ERK signaling in LX-2 cells following RGS5 siRNA treatment (Fig. 8A,B). ET-1 induced Rho activation in HSCs has been shown to enhance migration *in vitro* [84], and inhibition of ROCK improves fibrosis in choline deficient diet fed rats [85]. Loss of RGS5-mediated inhibition of ROCK could be one mechanism behind the enhanced HSC activation and fibrosis observed in *Rgs5^LacZ/LacZ^* mice.

RGS5 inhibition of ET-1 signaling has potential in the treatment of liver fibrosis and cirrhosis. While animal models of cirrhosis benefit from ET-1 antagonism, patients risk liver failure [86] due the hepatotoxic effects these drugs. RGS5 inhibition of ET-1 signaling specifically in HSCs provides a novel avenue for future therapies. Enhancing the expression of RGS5 in HSCs could reduce their activation and subsequent development of fibrosis and cirrhosis. The anti-fibrotic effects of RGS5 overexpression could be validated with studies of chronic CCl_4_ administration to mice in which RGS5 expression is conditionally overexpressed in HSCs. High RGS5 expression would be expected to block ET-1 mediated signaling in HSCs, potentially reducing HSC activation, contraction, migration, and ultimately fibrosis.

## Conclusions

RGS5 is a marker of HSCs that is up-regulated as HSCs respond to injury. RGS5 controls HSC activation via inhibition of ET-1 signaling and reduction of ERK activation. The critical role of RGS5 in liver injury is demonstrated by enhanced HSC activation, hepatocyte ballooning, and fibrosis in CCl_4_ treated RGS5-deficient mice. RGS5-mediated control of GPCR signaling in the liver is a novel mechanism by which HSC activation can be controlled, and a potential target of therapeutic intervention for liver fibrosis.

## Supporting Information

Figure S1 **RGS5 is expressed in freshly isolated primary HSCs.** 1° HSC have astrocyte-like morphology. **A**. Isolated 1° HSCs have an astrocyte-like phenotype and store vitamin A in lipid droplets. **B**. Isolated 1° HSCs are characterized by high RGS5 expression and low Tie1 and CD68 expression, indicating there are few contaminating cells. Low PDGFRβ expression demonstrates the 1° HSCs are in a quiescent state.
(TIF)

Figure S2 **Co-localization of β-gal^+^ nuclei and GFAP staining does not change during injury**. Frozen sections of CCl_4_ injected mouse liver were immunofluorescently labeled with antibodies for β-gal and GFAP. Co-localization analysis using ImagePro showed that the fraction of β-gal^+^ cells that are β-gal^+^ and GFAP^+^ does not significantly change during the course of injury. n = 3 mice per group.
(TIF)

Figure S3 **Liver weight to body weight ratio is moderately elevated in** ***Rgs5^lacZ/lacZ^*** **mice at 96hr post CCl_4_ injury.** Body and liver weights of CCl_4_ injected mice were measured at time of sacrifice. *Rgs5^LacZ/LacZ^* mice had elevated liver to body weight ratio 96 h post injury. *Rgs5^+/+^* liver to body weight ratio was not significantly different from untreated mice. * p = .001, ‡ p = .06, n = 4–6.
(TIF)

Figure S4 **Hepatocyte clearing is not associated with lipid accumulation.** Oil red O staining of mouse liver 96 hr post CCl_4_ injection was used to assess lipid accumulation. **A**. Lipid droplets are visible around the site of injury in both *Rgs5^+/+^* **A**,**C** and *Rgs5^LacZ/LacZ^* **B,D** mice. Cleared hepatocytes are visible in **B**, distant from the site of injury and droplets of lipid. **C,D**. Oil red O staining is present, but there is no accumulation of lipid in hepatocytes. Scale bar is 100 µm.
(TIF)

Figure S5 **Hepatocyte clearing is not associated with glycogen accumulation.** Periodic acid schiff stain of frozen mouse liver 96 hr post CCl_4_ injection was used to assess glycogen accumulation. **A**. Hepatocytes appear normal in *Rgs5^+/+^* liver. Intense magenta staining labels glycogen in the hepatocytes. **B**. Cleared hepatocytes are visible in *Rgs5^LacZ/LacZ^* and glycogen staining is present in hepatocytes. High magnification of **C** *Rgs5^+/+^* and **D** *Rgs5^LacZ/LacZ^* liver show RGS5 that cleared hepatocytes do not contain accumulated glycogen. Scale bar is 100 µm.
(TIF)

Figure S6 **Cleared hepatocytes are not apoptotic.** TUNEL staining of mouse liver 96 hr post CCl_4_ injection was used to assess apoptosis. **A**. *Rgs5^+/+^* liver shows TUNEL minimal staining 96 hr post injection. **B**. Cleared hepatocytes are visible in *Rgs5^LacZ/LacZ^* liver 96 hr post CCl_4_. No difference in TUNEL staining is visible in cleared hepatocytes. **C**. DNAse I treated positive control shows intense staining. **D**. Uninjured *Rgs5^LacZ/LacZ^* has minimal TUNEL staining. Scale bar is 100 µm.
(TIF)
