# Supplementary figures and images for: Regulator of G-Protein Signaling-5 Is a Marker of Hepatic Stellate Cells and Expression Mediates Response to Liver Injury
Source: PLoS One. 2014 Oct 7;9(10):e108505. doi: 10.1371/journal.pone.0108505 (PMC4188519; doi:10.1371/journal.pone.0108505)

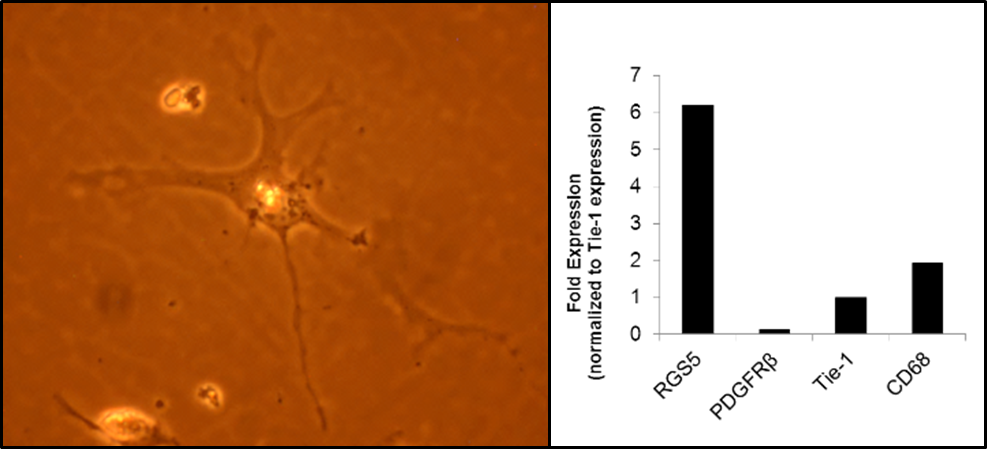

Supplement: Figure S1 — RGS5 is expressed in freshly isolated primary HSCs. 1° HSC have astrocyte-like morphology. A. Isolated 1° HSCs have an astrocyte-like phenotype and store vitamin A in lipid droplets. B. Isolated 1° HSCs are characterized by high RGS5 expression and low Tie1 and CD68 expression, indicating there are few contaminating cells. Low PDGFRβ expression demonstrates the 1° HSCs are in a quiescent state. (TIF) [file pone.0108505.s001.tif]

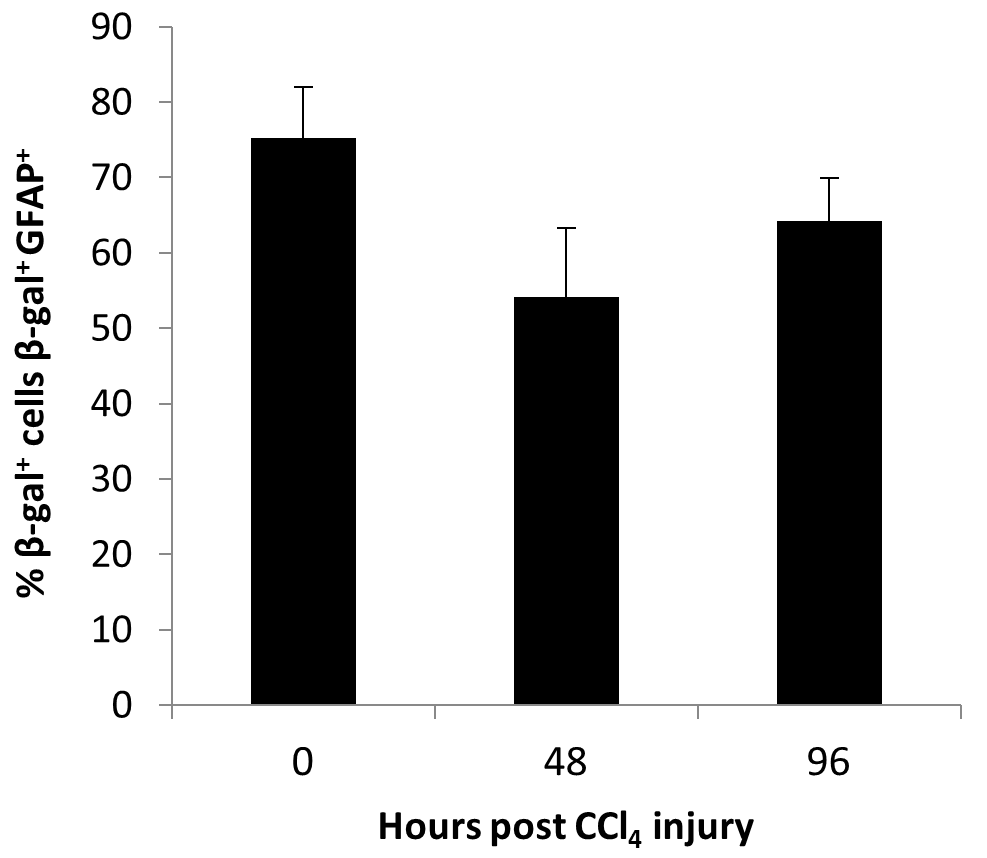

Supplement: Figure S2 — Co-localization of β-gal+ nuclei and GFAP staining does not change during injury. Frozen sections of CCl4 injected mouse liver were immunofluorescently labeled with antibodies for β-gal and GFAP. Co-localization analysis using ImagePro showed that the fraction of β-gal+ cells that are β-gal+ and GFAP+ does not significantly change during the course of injury. n = 3 mice per group. (TIF) [file pone.0108505.s002.tif]

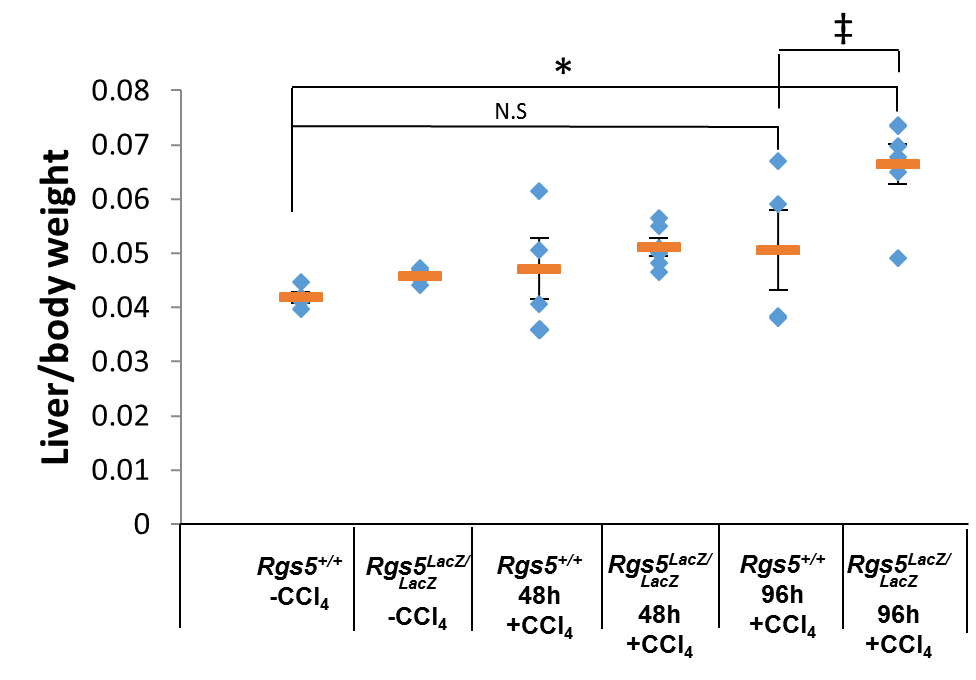

Supplement: Figure S3 — Liver weight to body weight ratio is moderately elevated in Rgs5lacZ/lacZ mice at 96hr post CCl4 injury. Body and liver weights of CCl4 injected mice were measured at time of sacrifice. Rgs5LacZ/LacZ mice had elevated liver to body weight ratio 96 h post injury. Rgs5+/+ liver to body weight ratio was not significantly different from untreated mice. * p = .001, ‡ p = .06, n = 4–6. (TIF) [file pone.0108505.s003.tif]

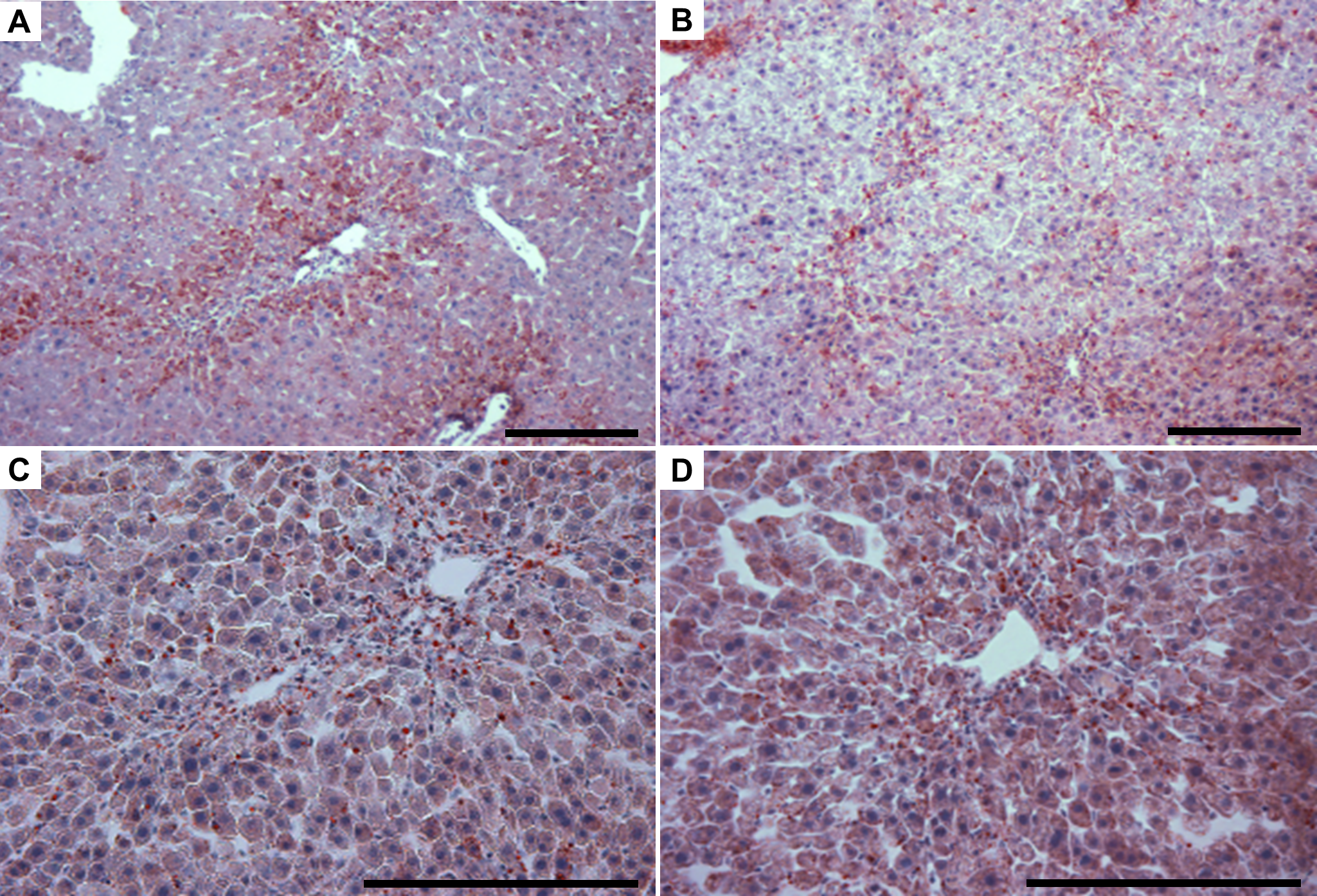

Supplement: Figure S4 — Hepatocyte clearing is not associated with lipid accumulation. Oil red O staining of mouse liver 96 hr post CCl4 injection was used to assess lipid accumulation. A. Lipid droplets are visible around the site of injury in both Rgs5+/+ A,C and Rgs5LacZ/LacZ B,D mice. Cleared hepatocytes are visible in B, distant from the site of injury and droplets of lipid. C,D. Oil red O staining is present, but there is no accumulation of lipid in hepatocytes. Scale bar is 100 µm. (TIF) [file pone.0108505.s004.tif]

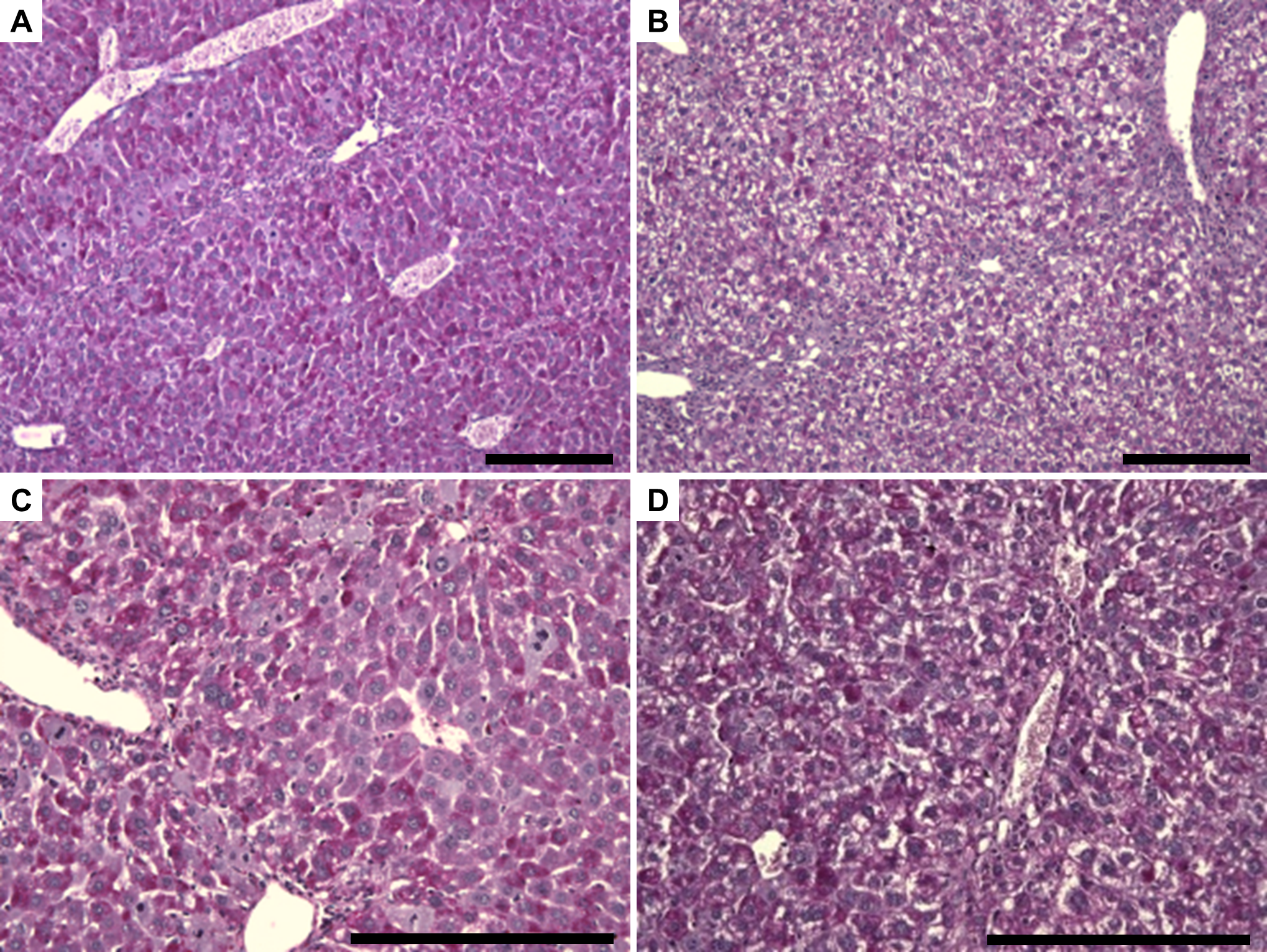

Supplement: Figure S5 — Hepatocyte clearing is not associated with glycogen accumulation. Periodic acid schiff stain of frozen mouse liver 96 hr post CCl4 injection was used to assess glycogen accumulation. A. Hepatocytes appear normal in Rgs5+/+ liver. Intense magenta staining labels glycogen in the hepatocytes. B. Cleared hepatocytes are visible in Rgs5LacZ/LacZ and glycogen staining is present in hepatocytes. High magnification of C Rgs5+/+ and D Rgs5LacZ/LacZ liver show RGS5 that cleared hepatocytes do not contain accumulated glycogen. Scale bar is 100 µm. (TIF) [file pone.0108505.s005.tif]

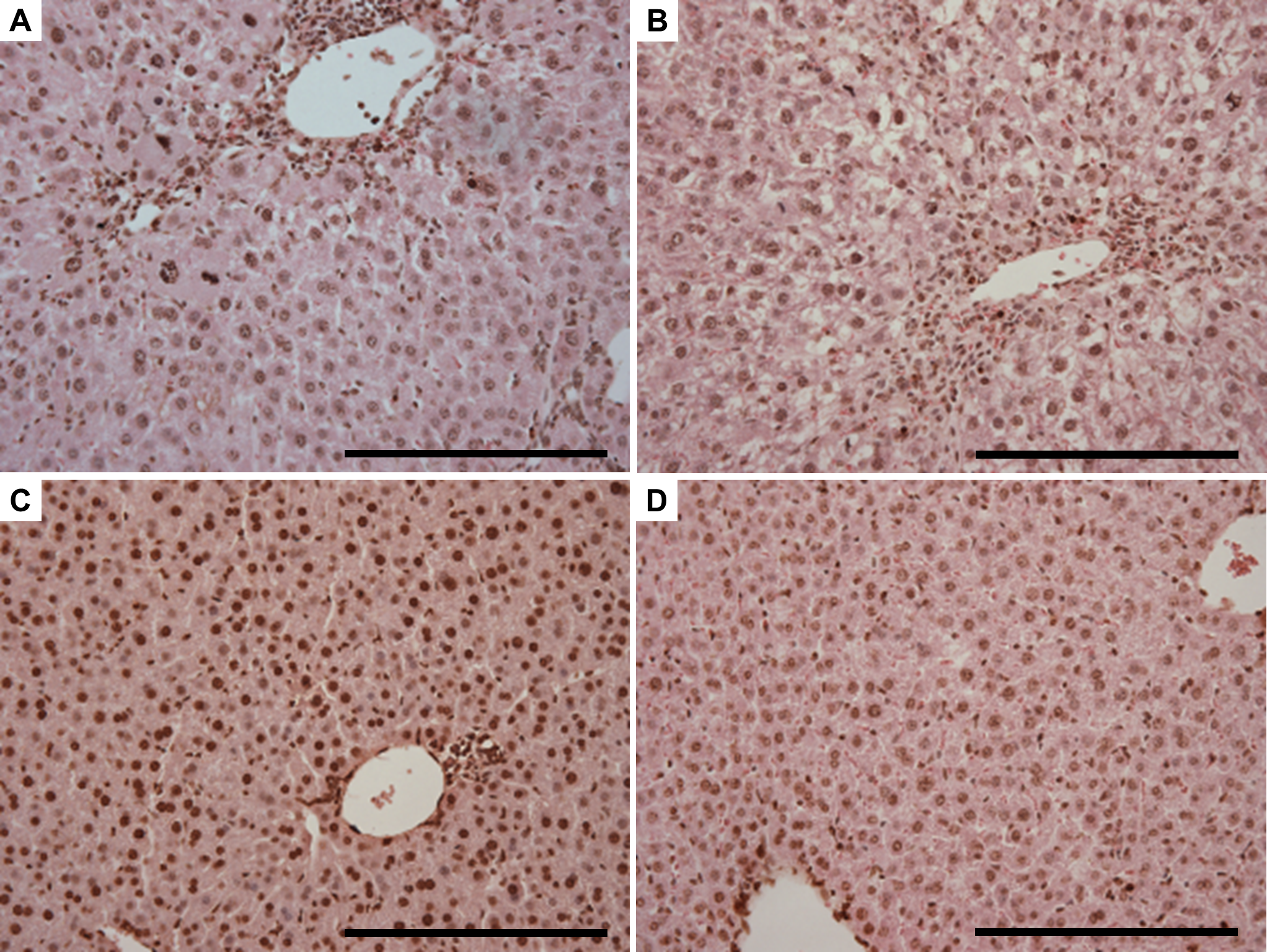

Supplement: Figure S6 — Cleared hepatocytes are not apoptotic. TUNEL staining of mouse liver 96 hr post CCl4 injection was used to assess apoptosis. A. Rgs5+/+ liver shows TUNEL minimal staining 96 hr post injection. B. Cleared hepatocytes are visible in Rgs5LacZ/LacZ liver 96 hr post CCl4. No difference in TUNEL staining is visible in cleared hepatocytes. C. DNAse I treated positive control shows intense staining. D. Uninjured Rgs5LacZ/LacZ has minimal TUNEL staining. Scale bar is 100 µm. (TIF) [file pone.0108505.s006.tif]
